# Supplementary material for: Predicting visual function by interpreting a neuronal wiring diagram
Source: Nature. 2024 Oct 2;634(8032):113–23. doi: 10.1038/s41586-024-07953-5 (PMC11446822; doi:10.1038/s41586-024-07953-5)
Supplement: Supplementary file 6 — CRF and ERF predictions for individual TmY4 and TmY9 cells. Analogous to Supplementary Data 3, but for TmY target types. Shown are the top four monosynaptic pathways, the strongest pathway passing through each of the top ten intermediary types (ranking from Extended Data Fig. 7), and the trisynaptic pathway Tm1–TmY–Dm3–TmY (see the section entitled Prediction of spatial normalization). [file 41586_2024_7953_MOESM6_ESM.zip › DataS4/TmY9qΓèÑ/720575940630573306.pdf]

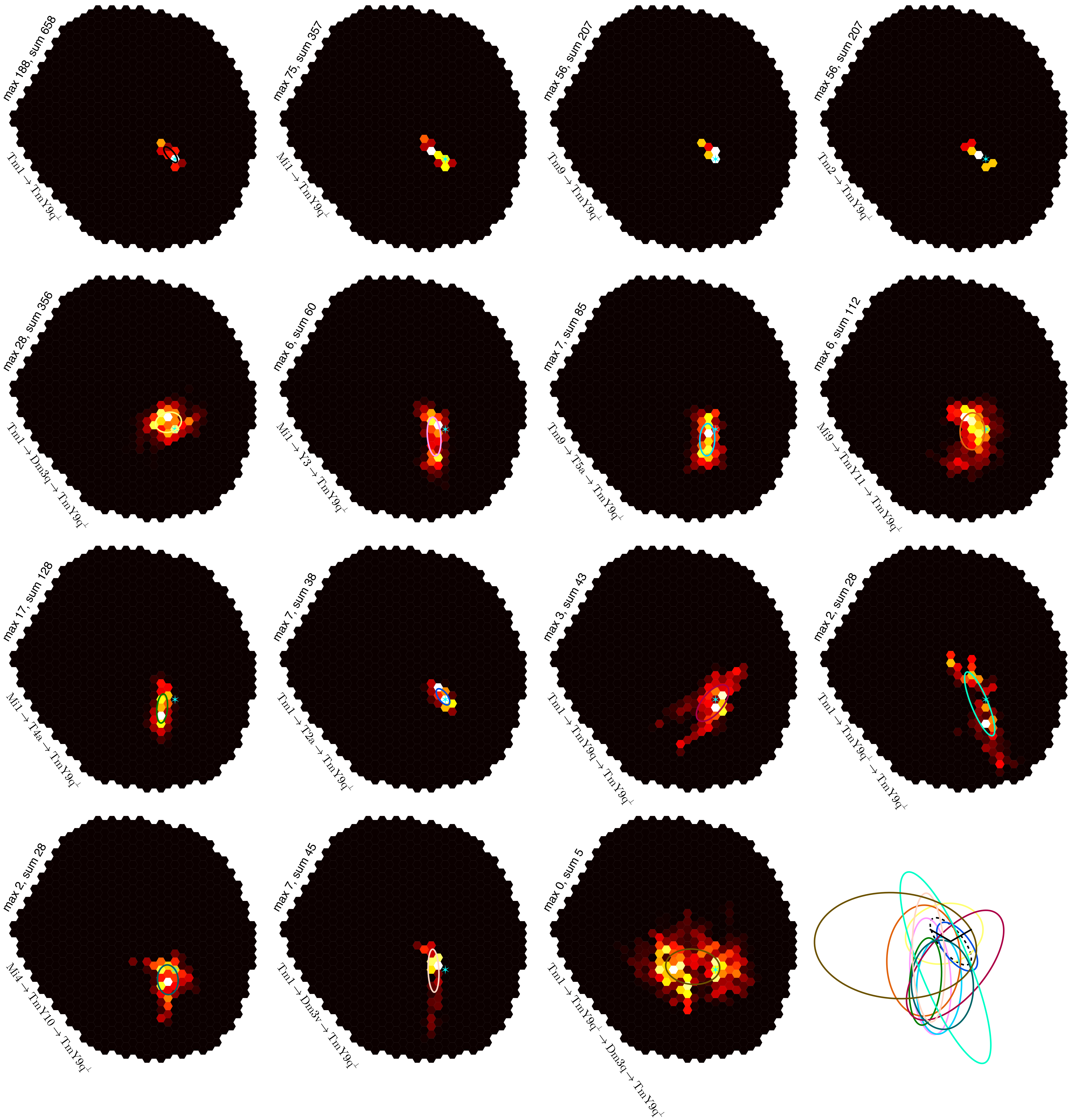
max 188, sum 658
Tm1 → TmY9q⊥
max 75, sum 357
Mi1 → TmY9q⊥
max 56, sum 207
Tm9 → TmY9q⊥
max 56, sum 207
Tm2 → TmY9q⊥
max 28, sum 356
Tm1 → Dm3q → TmY9q⊥
max 6, sum 60
Mi1 → Y3 → TmY9q⊥
max 7, sum 85
Tm9 → T5a → TmY9q⊥
max 6, sum 112
Mi9 → TmY11 → TmY9q⊥
max 17, sum 128
Mi1 → T4a → TmY9q⊥
max 7, sum 38
Tm1 → T2a → TmY9q⊥
max 3, sum 43
Tm1 → TmY9q⊥ → TmY9q⊥
max 2, sum 28
Tm1 → TmY9q⊥ → TmY9q⊥
max 2, sum 28
Mi4 → TmY10 → TmY9q⊥
max 7, sum 45
Tm1 → Dm3v → TmY9q⊥
max 0, sum 5
Tm1 → TmY9q⊥ → Dm3q → TmY9q⊥
